# From Broad-Spectrum Health to Targeted Prevention: A Review of Functional Foods in Chronic Disease Management

**DOI:** 10.3390/molecules31010103

**Published:** 2025-12-26

**Authors:** Xinyun Zhang, Qinghua Zeng, Wanchong He

**Affiliations:** 1 Shandong Key Laboratory of Applied Technology for Protein and Peptide Drugs, School of Pharmaceutical Sciences and Food Engineering, Liaocheng University, Liaocheng 252000, China; zhangxinyun929@163.com; 2 College of Agriculture and Life Sciences, Cornell University, Ithaca, NY 14850, USA

**Keywords:** functional foods, chronic disease prevention, food-medicine homology, mechanisms of action, societal acceptance

## Abstract

Chronic diseases, characterized by their high prevalence and protracted course, represent a paramount challenge to global public health, necessitating effective, evidence-based preventive strategies. While functional foods are widely recognized for their potential, a comprehensive synthesis elucidating their multitargeted mechanisms within a “food-medicine homology” framework and a clear trajectory from broad-spectrum health promotion to targeted intervention remains lacking. This review bridges this critical gap by systematically evaluating the scientific evidence and application potential of functional foods, with a specific focus on key bioactive compounds—β-glucan, omega-3 polyunsaturated fatty acids (PUFAs), dietary fiber, and catechins. We provide a critical analysis of how these components orchestrate synergistic effects at molecular, cellular, and systemic levels to counteract core pathological processes, including oxidative stress, chronic inflammation, metabolic dysregulation, and gut microbiota imbalance. Our unique contribution lies in integrating the ancient wisdom of food-medicine homology with modern multi-omics and evidence-based research, thereby proposing a refined nutritional intervention paradigm. The review offers critical insights into the convergent actions of these bioactives, their dose-response relationships substantiated by clinical meta-analyses, and the emerging role of gut microbiota-derived metabolites. Furthermore, this review also explores the emerging evidence for synergistic interactions among these key bioactives, proposing that their combined use may yield amplified and more network-based protective effects against chronic diseases through complementary mechanisms, aims to develop integrated prevention strategies targeting both cardiometabolic and neurodegenerative diseases. The integrated prevention strategies systematically connect mechanistic insights into bioactive compounds, evaluates the strength of clinical evidence, and examines the implications for regulatory standards and societal acceptance, thereby bridging the gap between basic science, clinical application, and public health policy. The “mechanism-to-evidence-to-regulation” framework in this review links molecular insights with clinical validation and regulatory implications, offering a holistic perspective rarely addressed in existing literature.

## 1. Introduction

Functional foods are natural or processed foods containing known or unknown bioactive compounds [1]. These compounds, at safe and effective doses, not only provide essential nutrition but also possess health-promoting, disease-preventing, and therapeutic effects against chronic diseases, extending beyond the scope of basic nutrition [2]. The bioactive compounds in functional foods exhibit diverse functions such as antioxidant, anti-inflammatory, lipid-lowering, blood glucose-regulating, cytoprotective, and neuroprotective activities, which constitute the key to their efficacy [3]. The mechanisms of action for functional foods are multifaceted, primarily including: regulating physiological rhythms and metabolism (e.g., blood glucose and lipid regulation), enhancing the body’s defense capabilities (e.g., antioxidant activity and immune modulation), preventing specific disease risks (e.g., cardiovascular disease and osteoporosis), promoting growth and development or maintaining specific physiological states (e.g., improving gut health and delaying aging), and restoring or maintaining internal homeostasis [4].

Current global research on functional foods exhibits distinct regional characteristics and technological integration trends. Domestically, research focuses intensively on the modern development of traditional medicinal food homology resources, employing multi-omics technologies to elucidate the mechanisms of action of bioactive compounds, and leveraging unique resources to develop novel functional factors; however, significant gaps remain in clinical evidence levels and functional factor delivery technologies [5]. Concurrently, international frontier research centers on precision nutrition interventions, integrating genomics, microbiome research, and real-time monitoring technologies, while leading innovation in delivery systems; regulatory frameworks in Europe, America, and Japan place greater emphasis on high-level scientific evidence, driving the approval of strict health claims certifications for components such as phytosterols and β-glucans [6].

The advancement of functional foods will be guided by the core principles of precision, efficacy, and scientific rigor. This entails integrating multi-omics analyses and real-time physiological monitoring technologies to achieve individualized dynamic formulation of functional factors and precise intervention; leveraging innovations in delivery systems such as nano-encapsulation and liposomes to significantly enhance the bioavailability and targeting of bioactive compounds; establishing high-level evidence-based frameworks grounded in large-scale cohort studies and real-world evidence to deeply elucidate the interaction mechanisms within the “food-microbiota-host” triad; concurrently, global regulatory harmonization efforts and the modernization of medicinal food homology resources will jointly drive industrial upgrading, ultimately realizing a scientific transition from broad-spectrum health promotion to targeted prevention of chronic diseases [7]. Furthermore, given the multifactorial nature of chronic diseases, nutritional strategies that combine multiple bioactive components may produce superior effects through synergistic interactions. This review therefore also evaluates the potential and evidence for synergy among the discussed functional food components, framing their integrated use as a promising multi-targeted approach [8].

In contrast to earlier reviews that predominantly summarize established knowledge on individual functional food components or focus on isolated disease pathways, this review offers several distinct and timely contributions. First, it provides a systematic and critical evaluation of the synergistic interactions among four key bioactive compounds—β-glucan, omega-3 PUFAs, dietary fiber, and catechins—a topic that has received limited integrated coverage in recent reviews despite growing experimental evidence. Second, this review prioritizes the inclusion of recent (primarily post-2020) preclinical and clinical studies, including emerging technologies such as structural modulation of β-glucans, metabolic engineering of catechin-enriched crops, and novel delivery systems, thereby updating the evidence base beyond traditional summaries. Third, and most importantly, it adopts a novel and comprehensive “mechanism-to-evidence-to-regulation” framework. This approach not only elucidates molecular and physiological mechanisms but also critically appraises the corresponding clinical evidence, and extends the discussion to how such evidence informs regulatory standards and societal acceptance. This end-to-end perspective bridges the gap between basic science, clinical application, and public health policy—a holistic integration seldom attempted in previous functional food reviews. Thus, this work moves beyond mere summation to present a forward-looking, integrative paradigm that highlights synergistic potential, incorporates contemporary research, and connects scientific discovery with translational and societal relevance.

## 2. Literature Search Methodology

The authors conducted a systematic literature search to ensure a rigorous and evidence-based foundation for this review. Primary searches were performed in the Web of Science Core Collection database for publications up to August 2025. The search strategy employed a combination of broad terms (“functional foods”, “chronic disease prevention”) and targeted queries for specific bioactive compounds (e.g., “beta-glucan AND cholesterol”, “omega-3 fatty acids AND neuroinflammation,” “dietary fiber AND diabetes”, “catechins AND antioxidant”).

The screening process involved two sequential stages. First, titles and abstracts were assessed against inclusion criteria focusing on peer-reviewed original research, systematic reviews, and meta-analyses relevant to the mechanisms or efficacy of the discussed bioactives in chronic disease contexts. Non-peer-reviewed literature and studies unrelated to chronic disease outcomes were excluded. Subsequently, the full texts of selected articles were evaluated for methodological quality and relevance, with particular attention to study design, sample size, and translational value of findings in human versus preclinical studies.

This structured approach enabled a critical synthesis of the literature, allowing the authors to transparently evaluate and present the varying strength of evidence—from robust clinical meta-analyses to promising preclinical data—that underpins the discussions throughout this review.

## 3. The Role of Functional Foods in Chronic Disease Prevention

Functional foods exert beneficial modulation on specific physiological functions in the human body, thereby promoting health status or reducing disease risk. Against the backdrop of chronic diseases such as cardiovascular disease, type II diabetes, and neurodegenerative diseases increasingly becoming a global public health burden, functional foods—through their rich diversity of bioactive compounds—act synergistically at molecular, cellular, and systemic levels on key pathophysiological processes including antioxidant defense, inflammation regulation, metabolic homeostasis maintenance, gut microbial homeostasis, and cytoprotection. This results in multi-target, network-based protective mechanisms, thereby playing a significant role as a nutritional intervention in preventing and delaying the onset and progression of major chronic diseases like cardiovascular disease, type II diabetes, and neurodegenerative diseases. Research into their mechanisms of action provides a robust scientific foundation for food-based precision nutrition strategies [9]. The following lists several bioactive components commonly found in functional foods and comparative position (as shown in Table 1), aslo frequently studied for their association with chronic disease prevention:

### 3.1. β-Glucan

β-Glucan is widely present in cereals such as oats and barley, as well as edible mushrooms including shiitake and lingzhi (Ganoderma lucidum) [10]. Its primary functions include: assisting in the reduction of cholesterol (particularly “bad” cholesterol LDL), stabilizing postprandial blood glucose levels, enhancing immune function, promoting the growth of beneficial gut bacteria, and increasing satiety [11]. Recent research has further elucidated its multifaceted physiological functions, demonstrating significant advances particularly in the precise modulation of gut microbiota, neuroprotection, and antitumor activity [12]. Upon ingestion, β-glucan rapidly hydrates within the digestive tract to form a highly viscous gel, significantly delaying gastric emptying and impeding chyme diffusion. This physical barrier effect effectively slows the transport rate of glucose to the intestinal epithelium, thereby improving the postprandial glycemic response and enhancing insulin sensitivity. More significantly, the abundant hydroxyl groups within its molecular structure strongly bind primary bile acids in the gut through hydrogen bonding and hydrophobic interactions, disrupting the enterohepatic circulation of bile acids and facilitating their fecal excretion. To compensate for the depletion of the bile acid pool, the liver is stimulated to upregulate the synthesis of new bile acids from cholesterol, directly depleting hepatic cholesterol reserves. Furthermore, undigested β-glucan serves as a prebiotic substrate fermented by colonic microbiota, producing short-chain fatty acids (SCFAs). Among these, butyrate serves as the primary energy source for colonic epithelial cells and maintains gut barrier integrity; propionate, upon absorption into the liver, inhibits the activity of HMG-CoA reductase, the rate-limiting enzyme in cholesterol synthesis [13]. Notably, novel structural modulation technologies are advancing the precision of prebiotic effects: the acid hydrolysis degradation technology for hyperbranched β-glucans (e.g., PTR-HBG derived from Pleurotus tuber-regium) developed by Shi Group (0.1–0.2 mol/L HCl, 90 °C), achieves controlled molecular weight reduction via a first-order kinetic model, with its degradation products significantly enhancing SCFA production and the proliferation efficiency of probiotics such as *Bifidobacterium* spp. in in vitro fermentation studies (2025) [14]. This structural tailoring approach exemplifies a promising direction for enhancing the prebiotic efficacy of β-glucans. By moving beyond native polymers, such strategies could enable the development of next-generation functional ingredients with predictable and potent microbiota-modulating effects, potentially overcoming the variability associated with natural sources.

In cardiovascular disease prevention, β-glucan’s multiple mechanisms act synergistically on core risk factors for atherosclerosis. The cholesterol-lowering effect represents its most central mechanism: to replenish cholesterol for bile acid synthesis, the liver significantly upregulates the expression and activity of low-density lipoprotein receptors (LDL-R), thereby accelerating the uptake and clearance of circulating low-density lipoprotein cholesterol (LDL-C), leading to a significant reduction in plasma total cholesterol (TC) concentration. Concurrently, the viscous gel it forms physically impedes dietary cholesterol absorption, while propionate generated from microbial fermentation further suppresses endogenous cholesterol synthesis in the liver [15]. This core mechanism is substantiated by extensive clinical research. For instance, a meta-analysis encompassing 21 randomized controlled trials (RCTs, *n* = 1120) confirmed that in individuals with mild hypercholesterolemia, daily intake of ≥3 g β-glucan for ≥3 weeks significantly reduces serum TC (mean difference [MD] = −0.27 mmol/L, 95% CI: −0.33, −0.21, *p* < 0.001) and LDL-C (MD = −0.26 mmol/L, 95% CI: −0.32, −0.20, *p* < 0.001), with the lipid-lowering efficacy potentially modified by food matrix (solid products may confer greater efficacy). Another RCT in patients with hypercholesterolemia (*n* = 75) demonstrated that daily intake of 6 g concentrated oat β-glucan for 6 weeks significantly reduced TC (−0.3 ± 0.1 mmol/L) and LDL-C (−0.3 ± 0.1 mmol/L, *p* = 0.03 vs. control [glucose]) compared to the control group (glucose). A systematic review and meta-analysis focusing on barley-derived β-glucan (including 14 RCTs, *n* = 615) similarly concluded that consumption of a diet rich in barley β-glucan (median dose ~6.5–6.9 g/day for a median duration of 4 weeks) significantly reduces LDL-C (MD = −0.25 mmol/L, 95% CI: −0.30, −0.20) and non-high-density lipoprotein cholesterol (non-HDL-C) (MD = −0.31 mmol/L, 95% CI: −0.39, −0.23), supporting the application of barley as a functional food in cardiovascular risk management [16]. While these meta-analyses and RCTs robustly confirm the lipid-lowering effect, the observed heterogeneity in effect sizes underscores the importance of context. The modifying role of the food matrix, molecular weight, and individual gut microbiota composition suggests that future applications may benefit from a more personalized approach, matching specific β-glucan formulations to individual consumer phenotypes for optimized outcomes.

The improvement in glycemic homeostasis is achieved by delaying glucose absorption, thereby mitigating glucotoxic stress on the vascular endothelium induced by postprandial hyperglycemia and hyperinsulinemia, and reducing the risk of insulin resistance [17]. SCFA (produced by β-glucan)-enhanced intestinal barrier function reduces the translocation of endotoxins (e.g., LPS) into the bloodstream, mitigating systemic low-grade inflammation induced by metabolic endotoxemia, collectively improving vascular endothelial function [18]. Notably, β-glucan’s modulation of gut microbiota structure constitutes a fundamental basis for its SCFA-mediated effects, exhibiting molecular weight dependency. A randomized controlled crossover study (*n* = not specified, employing a 5-week intervention + 4-week washout period) found that daily supplementation with 3 g high-molecular-weight (HMW) barley β-glucan significantly increased the abundance of Bacteroidetes phylum and Bifidobacterium genus while decreasing the abundance of Firmicutes phylum and Dialister genus. This specific microbial shift pattern was significantly correlated with improvements in cardiovascular disease risk markers (e.g., BMI, waist circumference, blood pressure, triglycerides), while low-molecular-weight (LMW) β-glucan showed no comparable effects [19]. This clear demonstration of molecular-weight-dependent microbial shifts is a critical insight. It moves the field from considering β-glucan as a uniform prebiotic to recognizing it as a class of molecules with distinct structure-function relationships. This knowledge is pivotal for designing functional foods aimed at targeting specific microbial populations or metabolic outcomes [20]. Recent preclinical research suggests a potential extension of this mechanism to neurodegenerative disease intervention: She Group demonstrated in an AD mouse model that highland barley β-glucan combined with Lactobacillus remodels the gut microbiota, reducing β-amyloid deposition by 39% and P-tau protein expression via the gut-brain axis, while repairing synaptic damage and significantly improving cognitive function (2025) [21]. This preclinical study powerfully illustrates the potential of combining prebiotics (β-glucan) with probiotics (*Lactobacillus*) for multi-target intervention in complex diseases like AD. However, these promising findings are derived from an animal model and require validation in human trials to assess their translational relevance. It provides a compelling rationale for investigating symbiotic formulations in human trials, particularly for conditions where systemic inflammation and gut-brain axis dysfunction are implicated.

In summary, β-glucan reduces the risks of hypercholesterolemia, hyperglycemia, and chronic inflammation through multiple targets: driving LDL-C(via upregulating LDL-R activity), inhibiting cholesterol absorption (through physical barrier formation) and synthesis (via propionate action), optimizing glycemic regulation (by delaying glucose absorption), and activating an SCFA-mediated anti-inflammatory network (modulating microbiota, producing SCFAs, exerting anti-inflammatory effects, and enhancing barrier function). Clinical evidence, including RCTs and meta-analyses, consistently demonstrates that adequate intake (typically ≥3 g/day) of oat- or barley-derived β-glucan effectively reduces TC and LDL-C levels. Simultaneously, its prebiotic properties induce beneficial alterations in gut microbiota (particularly with HMW variants), and the consequent increased production of SCFAs (especially butyrate) constitutes another critical dimension of its cardiovascular protective effects. Therefore, β-glucan represents a functional dietary ingredient with a robust scientific foundation for cardiovascular disease prevention.

### 3.2. Omega-3 Polyunsaturated Fatty Acids

Omega-3 polyunsaturated fatty acids (ω-3 PUFAs), particularly docosahexaenoic acid (DHA) and eicosapentaenoic acid (EPA), are critical structural and functional components of neural cells. Primarily found in fatty fish (e.g., salmon, mackerel, sardines) [22], walnuts, flaxseeds, chia seeds, and certain plant oils (e.g., flaxseed oil, canola oil), they are essential for maintaining cardiovascular health, supporting brain function, and preserving central nervous system homeostasis [23,24,25].

**Figure 1:**
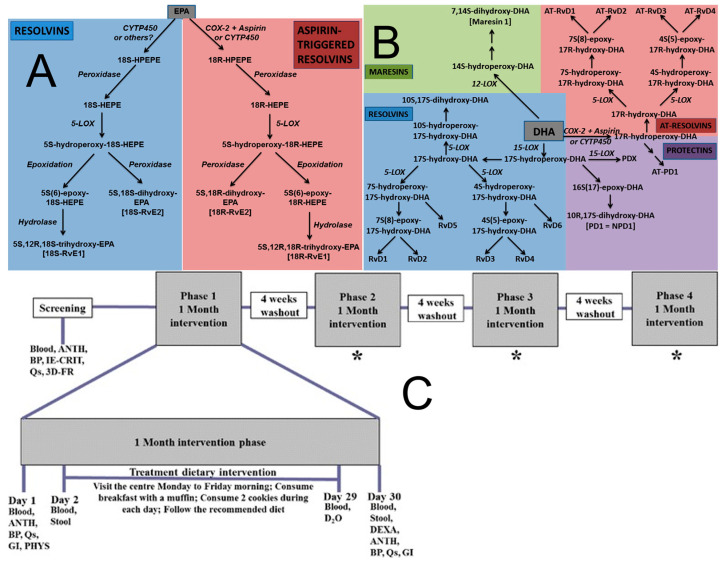
Omega-3 Fatty Acid Metabolism and Specialized Pro-resolving Mediators. (**A**) Outline of the pathways of resolvin and aspirin—triggered resolvin biosynthesis from eicosapentaenoic acid (EPA). COX, cyclooxygenase; CYIP450, cytochrome; HEPE, hydroxyeicosapentaenoic acid; HPETE, hydroperoxyeicosapentaenoic acid; LOX, lipoxygenase. Note: not all intermediates and enzymes are shown [38]. (**B**) Outline of the pathways of resolvin, protectin, aspirin-triggered resolvin and protectin, and maresin biosynthesis from docosahexaenoic acid (DHA). AT-Rv, aspirin-triggered resolvin; COX, cyclooxygenase; CYIP450, cytochrome P450 enzymes; LOX, lipoxygenase; PDX, protectin DX (10S,17S-dihydroxy-DHA) [38]. (**C**) Schematic representation of the experimental protocol. Blood 12-h fasted blood collection, ANTH anthropometric measurements, BP blood pressure, IE-CRIT inclusion and exclusion criteria, QS questionnaires (fish and seafood consumption, concomitant medications, adverse events, physical activity), 3D-FR 3-day food record collection, DEXA dual-energy X-ray absorptiometry, PHYS physician examination, GI gastrointestinal questionnaires, STOOL stool sample collection, D_2_O deuterated water, * procedures are the same as in phase 1 [30].

In the prevention of neurodegenerative diseases, ω-3 PUFAs (particularly DHA and EPA) are hypothesized to exert neuroprotective effects through multi-target, multi-level mechanisms that have been largely elucidated from cell-line and animal studies. While some clinical trials support cognitive benefits in specific populations, the translation of these mechanistic insights to broad chronic disease prevention requires further clinical validation [26]. Firstly, by maintaining the integrity of the neuronal membrane lipid microenvironment, DHA contributes to reducing the processing of the β-amyloid precursor protein (APP) toward the amyloidogenic pathway. It may also directly interfere with Aβ oligomer formation and its toxic aggregation on neuronal membranes, while simultaneously promoting microglial clearance of Aβ [27]. Regarding Tau protein pathology, DHA and its derivative neuroprotectin D1 (NPD1) have been demonstrated to inhibit the excessive activation of kinases such as glycogen synthase kinase-3β (GSK-3β), thereby reducing the pathological hyperphosphorylation of Tau protein and the formation of neurofibrillary tangles [28]. Secondly, the potent anti-inflammatory and pro-resolving actions of DHA and EPA are crucial, as shown in Figure 1A,B: they effectively inhibit the polarization of microglia towards the pro-inflammatory M1 phenotype, reduce the production of reactive oxygen species (ROS) and reactive nitrogen species (RNS), block the assembly and activation of the NLRP3 inflammasome, and promote the transition of inflammation to the resolution phase, thereby mitigating the persistent neuronal damage caused by chronic neuroinflammation [29]. In vitro studies provide support for this mechanism: for instance, investigations using the BV-2 microglial cell model to explore the impact of different ω-6/ω-3 fatty acid ratios on neuroinflammation revealed that specific fatty acid compositions and ratios (e.g., 2:1 ω-6:ω-3) significantly reduced cell viability and modulated the cytokine secretion profile (e.g., decreased tumor necrosis factor-alpha (TNF-α)), suggesting a potential anti-inflammatory effect. Conversely, a high ω-6 ratio (15:1) induced elevated IL-17 and decreased IL-4 and IL-10, revealing the complexity of fatty acid regulation of microglial activation and cytokine networks. This intricate interplay significantly influences neuroinflammation and potentially neurodegenerative progression [30]. Such cell-based findings elucidate possible pathways but must be interpreted with the caveat that they represent a simplified system compared to the complex human brain environment. Thirdly, ω-3 PUFAs constitute an essential component of the endogenous antioxidant defense system. They directly quench free radicals and enhance the activity of antioxidant enzymes such as superoxide dismutase (SOD) and glutathione peroxidase (GPx), protecting neurons from oxidative stress damage [31]. Fourthly, DHA promotes the clearance of damaged organelles and misfolded proteins by upregulating the expression of autophagy-related genes and activating deacetylases such as SIRT1, thereby maintaining proteostasis. Furthermore, ω-3 PUFAs improve cerebrovascular function, enhance blood-brain barrier integrity, optimize cerebral blood flow perfusion and energy metabolism, providing optimal microenvironmental support for neurons. Notably, the cardiovascular benefits of ω-3 PUFAs may indirectly support neuroprotection. For example, as shown in Figure 1C, a randomized single-blind crossover trial in individuals with metabolic syndrome (MetS)—a population at high risk for neurodegenerative diseases—evaluated the effects of a combined food product rich in high-oleic canola oil (HOCO)-DHA (providing 3 g/day DHA) and high-molecular-weight barley β-glucan (4.36 g/day) [32]. Although the primary endpoints were cardiovascular disease risk factors (e.g., lipid profile, inflammatory status, blood pressure), its effects on improving vascular endothelial function, attenuating systemic inflammation and oxidative stress are crucial for maintaining cerebral blood perfusion and reducing the risk of neurovascular unit damage. This provides important evidence for understanding how ω-3 PUFAs reduce neurodegenerative risk by improving the systemic metabolic environment [33]. Innovative preclinical research in 2025 on C9orf72 mutation-associated frontotemporal dementia (FTD) demonstrated that increasing brain omega-3 content extended survival in model organisms and patient-derived cells [34]. This groundbreaking preclinical study highlights a novel potential mechanism and target; however, its therapeutic implications for human neurodegenerative diseases await testing in clinical trials [34]. It highlights the potential of omega-3 PUFAs as a component of precision nutrition strategies for specific patient subpopulations, moving the field towards genetically informed nutritional interventions [35].

Collectively, these mechanisms indicate that adequate ω-3 PUFA intake is considered beneficial for maintaining neuronal network function, delaying cognitive decline, and reducing the risk of specific neurodegenerative pathologies [36]. This protective effect finds some support in clinical research. For instance, intervention studies in individuals at high risk for AD or with mild AD (e.g., the ADvance trial) suggest that high-dose DHA supplementation (typically ≥2 g/day), particularly during early disease stages or in specific genetic backgrounds (e.g., APOE ε4 non-carriers), may induce favorable changes in cerebrospinal fluid Aβ42 levels and p-tau protein, or attenuate the decline rate in specific cognitive domains (e.g., memory). Similarly, in Parkinson’s disease (PD) research, EPA supplementation (e.g., 1–2 g/day) has been associated with slowed motor symptom progression and improved quality-of-life scores. The underlying mechanisms may involve mitigation of neuroinflammation in the nigrostriatal pathway, reduction of peripheral and central inflammatory markers (e.g., IL-6, TNF-α), and counteraction of oxidative stress. Nevertheless, the clinical efficacy of ω-3 PUFAs is not consistently observed across all studies and may be significantly modulated by multiple factors, including individual genetic background (e.g., APOE genotype), baseline nutritional status, dose and form of intake, intervention stage (prevention versus treatment), and disease severity [37].

The heterogeneity in clinical trial outcomes regarding the efficacy of ω-3 PUFAs, particularly in neurodegenerative and cardiovascular contexts, necessitates a deeper exploration of the underlying causes. These discrepancies complicate the formulation of broad public health recommendations. The key factors contributing to this variability are multifaceted and often interact, as summarized in Table 2. The synthesis of factors in Table 2 is crucial for interpreting the conflicting literature. It argues compellingly that the inconsistent results are not necessarily evidence of ineffectiveness, but rather of a complex, modifiable effect. This reframes the research question from “Do omega-3s work?” to “In whom, under what conditions, and at what dose do omega-3s work?”—a fundamental shift towards precision nutrition.

The initial omega-3 PUFA status of participants is a critical determinant. Trials enrolling individuals with low baseline levels often demonstrate significant benefits upon supplementation, whereas those involving populations with adequate or high baseline intake (e.g., regular fish consumers) may show minimal effects. This “ceiling effect” suggests that supplementation primarily benefits those with deficiencies or suboptimal intake.

Genetic polymorphisms significantly influence individual response. The APOE ε4 allele, a major genetic risk factor for Alzheimer’s disease, is consistently associated with a blunted response to DHA supplementation. APOE ε4 carriers exhibit impaired DHA incorporation into the brain and altered lipid metabolism, which may underlie the reduced efficacy observed in this subgroup. Furthermore, polymorphisms in the fatty acid desaturase (FADS) gene cluster affect the efficiency of converting plant-based alpha-linolenic acid (ALA) to EPA and DHA, influencing an individual’s reliance on pre-formed EPA and DHA from the diet or supplements.

Methodological aspects play a substantial role. Trial duration is crucial; studies of insufficient length may not capture the slow, modulative effects of omega-3 PUFAs on chronic diseases. The dose and formulation are equally important. Many early trials used doses now considered sub-therapeutic (<1 g/day EPA + DHA). Moreover, the ratio of EPA to DHA and the chemical form (e.g., triglyceride vs. ethyl ester) can affect bioavailability and bioactivity, contributing to inconsistent findings across studies.

The timing of intervention relative to disease progression is paramount. Omega-3 PUFAs are likely most effective in the primary prevention phase or very early stages of disease, acting to maintain cellular health and resilience. Initiating supplementation after significant neuronal loss or advanced atherosclerotic plaque formation has occurred may yield limited clinical benefits. The specific population studied (e.g., age, sex, overall health status) also introduces variability.

The inconsistent clinical evidence underscores the limitation of a one-size-fits-all approach to omega-3 PUFA recommendations. Generalized advice may be ineffective and inefficient. Instead, a shift towards precision nutrition strategies is warranted. Future public health guidelines could be stratified, emphasizing higher intake targets for populations with low baseline status, specific genetic backgrounds (e.g., recommending earlier and higher intake for APOE ε4 non-carriers in the context of cognitive health), or those at high risk due to comorbidities. Furthermore, recommendations should specify effective doses and consider the formulation for specific health outcomes. Ultimately, generating robust evidence for stratified guidelines requires future clinical trials to be designed with these modulating factors in mind, employing enrichment strategies for responsive populations and longer intervention periods to adequately assess preventive potential.

### 3.3. Dietary Fiber

Dietary fiber, defined as plant-derived carbohydrate polymers, is primarily found in plant-based foods such as whole grains (e.g., oats, brown rice), vegetables (e.g., broccoli, spinach), fruits (e.g., apples, bananas with peel), legumes (e.g., soybeans, lentils), nuts, and seeds. Its principal functions include aiding digestion, preventing constipation, increasing satiety, regulating blood glucose and cholesterol levels, and promoting gut health [39,40,41]; Systematic reviews and meta-analyses of large-scale randomized controlled trials (encompassing 28 studies, *n* = 1148–1394 patients) confirm that viscous fiber supplements (e.g., psyllium, guar gum, β-glucan, konjac; median dose 13.1 g/day), as adjuncts to conventional therapy, significantly improve key glycemic parameters including glycated hemoglobin and fasting blood glucose in type II diabetes patients [42]. Insoluble dietary fiber accelerates intestinal transit by increasing fecal bulk and water retention capacity. Notably, insoluble cereal fibers (e.g., wheat- or oat-derived) demonstrate significant preventive potential for type II diabetes in epidemiological and intervention studies. Large prospective cohort studies (such as the EPIC-InterAct study, *n* = 11,559 cases + 15,258 controls) and multiple meta-analyses consistently indicate that higher cereal fiber intake is associated with a significantly reduced risk of type II diabetes, with this association exhibiting dose dependency: for instance, a risk ratio (RR) of approximately 0.75 per 10 g/day increment in cereal fiber intake, or an RR of ~0.94 per 2 g/day increment [43]. This protective effect may partially originate from their capacity to modulate bile acid metabolism, as insoluble cereal fibers mechanically bind bile acids, promoting their excretion and thereby contributing to reduced plasma cholesterol levels and inhibition of dietary fat absorption, ameliorating dyslipidemia in type II diabetes patients.

**Figure 2:**
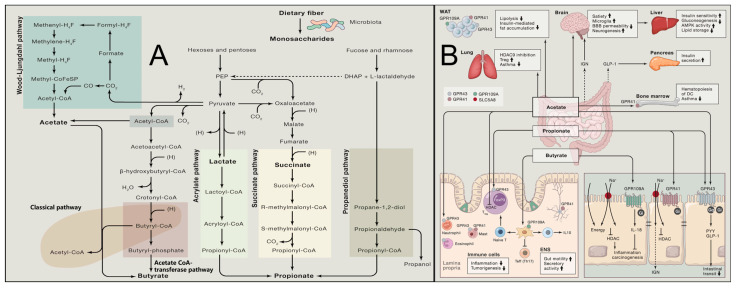
Gut Microbial Fermentation of Dietary Fibers and Systemic Actions of Short-Chain Fatty Acids. (**A**) Known pathways for biosynthesis of SCFAs from carbohydrate fermentation and bacterial cross-feeding [50]. (**B**) Mechanism of action of microbially produced SCFAs [50].

Metabolically, dietary fiber serves as the primary fermentation substrate for the colonic microbiota, generating SCFAs (including acetate, propionate, and butyrate), as shown in Figure 2A,B [44]. Emerging preclinical research further reveals that specific high-fiber formulations (e.g., CF30m, partially substituting macronutrients with cellulose) significantly activate key longevity and metabolic regulatory pathways such as AMPK/SIRT1 via SCFAs in model systems, thereby improving glucose and lipid metabolism and appearing to mimic some beneficial effects of caloric restriction independent of body weight changes [45]. Whether these specific pathway activations and metabolic mimics translate directly to long-term human health benefits requires confirmation in human intervention studies. Furthermore, insoluble fibers from select botanical sources exhibit unique metabolic regulatory potential: cabbage-derived insoluble fiber (CIDF) was demonstrated (2025) to effectively reduce cholesterol levels and ameliorate insulin resistance by modulating the “Bilophila-acetate axis” [46]. However, the understanding of mechanisms underlying traditionally considered poorly fermentable insoluble cereal fibers is evolving. Recent evidence indicates they may ameliorate insulin resistance by modulating blood amino acid metabolic profiles through effects on dietary protein digestion or absorption, while preventing excessive activation of the mammalian target of rapamycin (mTOR)/S6 kinase 1 (S6K1) signaling pathway [47]. This mechanism helps explain the association between high cereal fiber intake and lower diabetes risk in contexts of elevated protein consumption.

The preventive mechanisms of dietary fiber against type II diabetes involve multi-target synergistic actions. SCFAs produced through colonic fermentation enhance insulin sensitivity via multiple pathways: propionate activates GPR41/43 receptors in hepatic and peripheral tissues, inducing AMPK pathway activation that promotes GLUT4 translocation-mediated glucose uptake while suppressing hepatic gluconeogenesis; butyrate reinforces intestinal barrier function to reduce endotoxin translocation and directly inhibits inflammatory factor release from adipose tissue, blocking inflammation-mediated insulin resistance. Recent cell-based and molecular research provides a preclinical elucidation of the potential cancer-preventive mechanisms of SCFAs (particularly propionate and butyrate). While these regulated cellular homeostasis pathways are of mechanistic interest, their relevance to clinical chronic disease outcomes in humans awaits confirmation in population-based or intervention studies [48].

In obesity and metabolic disorder interventions, dietary fiber reduces energy intake by enhancing satiety, with long-term effects favoring weight control; its bile acid adsorption and excretion mechanism lowers circulating cholesterol levels, ameliorating dyslipidemia. Although weight control represents a significant mechanism, randomized controlled trials (e.g., the OptiFit trial) reveal that sustained high cereal fiber intake via supplementation (e.g., high-purity insoluble cereal fiber extracted from oat hulls: containing 70% cellulose, 25% hemicellulose, 3–5% lignin, administered twice daily for two years) can induce trends toward improved glycated hemoglobin and blood glucose concentrations—particularly in females—and suggest reduced type II diabetes incidence, even when no significant weight change difference exists between high-fiber and placebo groups. This strongly supports that the beneficial metabolic effects of high cereal fiber intake operate via mechanisms independent of weight alterations [49].

In summary, through integrated mechanisms including delaying nutrient absorption, SCFA-mediated metabolic and inflammatory regulation, incretin secretion, and maintenance of microbial homeostasis, dietary fiber plays a core protective role in the primary prevention of T2DM. Robust epidemiological evidence (such as meta-analyses encompassing over 300,000 participants and 8500 cases) and a growing body of intervention studies (e.g., the OptiFit trial) collectively establish that cereal fiber, particularly insoluble cereal fiber, exerts a prominent effect among various dietary fibers in reducing the risk of type 2 diabetes. Significant advances have also been made in value-added utilization technologies for fiber resources; for instance, ultra-efficient solubilization techniques for fibers derived from sources such as Ganoderma lucidum, Auricularia heimuer (black wood ear fungus), and tea leaves significantly enhance the release rate and palatability of soluble dietary fiber (SDF) (reducing molecular weight below 50 kDa), resolving textural coarseness issues and paving the way for developing more acceptable and effective fiber supplements or fortified foods. Substantially increasing population-level intake of cereal fiber (e.g., through safe and effective fiber supplements or fortified foods) is anticipated to become an important public health strategy for preventing type 2 diabetes. The translation of this strong epidemiological evidence into effective public health strategy faces the challenge of dietary adherence. The development of palatable, convenient, and effective fiber-fortified foods or supplements, as alluded to with advanced solubilization techniques, is therefore not merely a technological pursuit but a critical translational step for realizing the population-level benefits demonstrated in cohorts like EPIC-InterAct.

### 3.4. Potent Antioxidant and Anti-Inflammatory Properties of Catechins

Catechins, the core components of tea polyphenols, are predominantly found in foods such as tea (especially green tea), cocoa beans, dark chocolate, apples, grapes, and berries (e.g., strawberries, blueberries). They primarily function to protect body cells, exert anti-inflammatory and antioxidant effects, and benefit heart health. Their exceptional antioxidant activity stems from their unique chemical structure featuring phenolic hydroxyl groups. These phenolic hydroxyl groups efficiently donate hydrogen atoms or electrons, directly quenching various ROS and effectively interrupting free radical chain reactions, thereby inhibiting oxidative damage to biomacromolecules. Furthermore, catechins chelate transition metal ions, preventing their participation in catalytic ROS-generating pathways such as the Fenton reaction, thus reducing oxidative stress at its source [51]. Recent studies further reveal a breakthrough in addressing the bottleneck of poor in vivo stability of catechins: acetylated planar catechin analogs employ a “protection-release” strategy. The acetyl modification forms a molecular protective shield, maintaining stability during delivery. Upon reaching cancer cells, they are activated by esterases, releasing a superoxide radical scavenging capacity enhanced 10-fold compared to natural catechins. This preclinical strategy significantly augments targeted toxicity in experimental models, providing a novel paradigm for the design of targeted anticancer drug delivery systems that warrants future investigation. More importantly, catechins exert potent indirect antioxidant effects through activation of the Keap1-Nrf2/ARE signaling pathway: catechins modify key cysteine residues on the Keap1 protein, facilitating the dissociation of nuclear factor erythroid 2-related factor 2 (Nrf2) from Keap1 and its subsequent nuclear translocation. Nrf2 then binds to antioxidant response elements (AREs), significantly upregulating the gene expression of a suite of endogenous antioxidant enzymes and phase II detoxifying enzymes, thereby systemically enhancing cellular antioxidant defense capacity [52]. This mechanism has found innovative application in agriculture—the Luo Group successfully developed “tea-infused rice” with endosperm-enriched functional components such as epicatechin by introducing key catechin biosynthesis genes from tea plants into rice through endosperm-specific metabolic engineering. This breakthrough not only provides a novel dietary approach for caffeine-sensitive individuals to ingest catechins through staple food but also demonstrates the technical feasibility of directed synthesis of high-value-added natural products in crops (as shown in Table 3) [53]. However, the long-term safety, bioavailability of the engineered catechins, and their subsequent health effects upon human consumption remain to be evaluated in dietary intervention studies.

**Table 3:** Comparison of active components in the endosperm of tea rice and traditional rice [54].

Core Component	Traditional Rice Endosperm	Tea Rice Endosperm
Catechins	Almost zero	Significantly enriched
Total curcumin content	Extremely low	The level of functional activity
Caffeine	No	No

**Figure 3:**
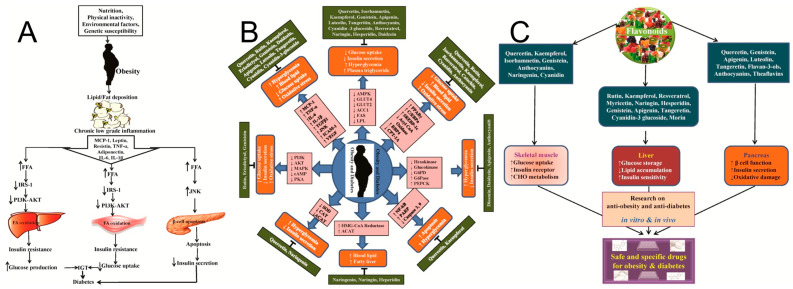
Pathophysiological Network of Cardiometabolic Diseases and Multi-Target Intervention by Dietary Polyphenols. (**A**) Schematic diagram of the link between obesity and diabetes as well as their effects in skeletal muscle, liver, and pancreas for stimulating different inflammatory cytokines, metabolic enzymes, and signaling pathways [60]. (**B**) Schematic presentation of molecular functions of different flavonoids with anti-obesity and anti-diabetic effects [60]. (**C**) Graphical presentation of anti-obesity and anti-diabetes effect of flavonoids and their subsequent effects in skeletal muscles, liver, and pancreas to induce glucose uptake, increase insulin secretion, and reduce oxidative damage and lipid accumulation [60].

In terms of anti-inflammatory effects, catechins exert significant actions through multi-target modulation of pro-inflammatory signaling pathways: they effectively inhibit the activation of the key transcription factor NF-κB by blocking IκB kinase phosphorylation, reducing IκBα degradation, and suppressing nuclear translocation and DNA-binding activity of the NF-κB subunit p65; simultaneously, catechins also inhibit the activation of MAPK and JAK-STAT pathways. These actions lead to significant downregulation of the gene expression and secretion of multiple pro-inflammatory cytokines and chemokines. Furthermore, catechins suppress inflammasome assembly and activation, reduce caspase-1-mediated maturation of interleukin-1β (IL-1β) and IL-18, and inhibit the expression of inducible nitric oxide synthase (iNOS) and cyclooxygenase-2 (COX-2), thereby reducing excessive production of inflammatory mediators such as nitric oxide and prostaglandin E2. Obesity has a great impact on all body functions, as shown in Figure 3A. Recent research reveals that catechins possessing C-3 galloyl and B-5 hydroxyl group structures (e.g., EGCG, ECG) exhibit marked structure-function specificity in anti-obesity effects—they specifically promote the proliferation of the probiotic Akkermansia muciniphila, modulate the LPS/insulin resistance signaling pathway, and establish a bidirectional “catechin-microbiota” amplification loop, effectively ameliorating high-fat diet-induced obesity, fatty liver, and glucose-lipid metabolic disorders [55].

Through the potent antioxidant and anti-inflammatory effects described above, catechins intervene at the molecular and cellular levels in the common pathophysiological basis of multiple chronic diseases—chronic low-grade inflammation and persistent oxidative stress—thereby demonstrating key mechanisms of action in preventing several major chronic diseases. In cardiovascular disease prevention, catechins protect LDL from oxidative modification and decreasing foam cell formation; their anti-inflammatory effects suppress vascular endothelial cell activation, adhesion molecule expression, and monocyte infiltration, improving endothelial function while inhibiting vascular smooth muscle cell proliferation and migration, collectively attenuating atherosclerotic progression. Simultaneously, their antioxidant effects help maintain myocardial mitochondrial function and mitigate ischemia-reperfusion injury. Robust epidemiological evidence supports this protective role: the large-scale 11-year Ohsaki cohort study in Japan found that individuals consuming ≥5 cups of green tea (rich in catechins) daily exhibited significantly lower cardiovascular disease mortality (particularly stroke) compared to those consuming <1 cup daily [56]. This effect is partially attributed to catechin-mediated improvements in vascular endothelial function and reductions in systemic oxidative stress markers (e.g., 8-OHdG, MDA). In the context of type 2 diabetes and metabolic syndrome, catechins regulate blood glucose by improving insulin sensitivity and suppressing the expression of key gluconeogenic enzymes in the liver, as shown in Figure 3B,C; their antioxidant and anti-inflammatory actions alleviate adipose tissue inflammation, reduce hepatic steatosis and inflammation, improve insulin resistance, and promote fatty acid oxidation and energy metabolism via AMPK pathway activation. RCTs provide direct evidence: a study in prediabetic subjects demonstrated that sustained high-dose catechin intervention (e.g., >500 mg EGCG daily) for 12 weeks resulted in significant decreases in fasting blood glucose, homeostatic model assessment of insulin resistance (HOMA-IR), and serum inflammatory markers (e.g., CRP, IL-6), alongside an increase in adiponectin levels [57]. This indicates that catechins delay diabetes progression through anti-inflammatory effects and amelioration of metabolic dysregulation. In neurodegenerative diseases, catechins cross the blood-brain barrier; their potent antioxidant capacity directly scavenges neurotoxic free radicals, inhibits Aβ amyloid aggregation and tau protein hyperphosphorylation, and suppresses aberrant activation of microglia and astrocytes along with the neurotoxic inflammatory factors they produce. This protects neurons from oxidative damage and inflammation-mediated apoptosis. Preclinical and preliminary clinical studies support their neuroprotective potential: for instance, in Alzheimer’s disease model animals, EGCG intervention significantly reduced cerebral Aβ plaque deposition and phosphorylated tau protein while improving cognitive function. A preliminary, small-scale human trial in individuals with mild cognitive impairment reported that intake of green tea catechin extract was associated with improvements in some cognitive measures and stabilized Aβ42 levels in cerebrospinal fluid. These exploratory findings warrant replication in larger, longer-term randomized controlled trials [58]. Furthermore, catechins exert deeper effects in chronic disease prevention by influencing gene expression through epigenetic regulation. Studies confirm that catechins (particularly EGCG), acting as potent inhibitors of DNA methyltransferases (DNMTs) and histone deacetylases (HDACs), can reverse aberrant epigenetic silencing or activation of genes associated with chronic inflammation and oxidative stress (e.g., pro-inflammatory cytokine genes, tumor suppressor genes). For instance, in cancer chemoprevention research, EGCG treatment has been demonstrated to induce promoter demethylation and restore expression of various tumor suppressor genes (such as p16INK4a and RARβ); in metabolic disease models, its epigenetic regulatory role has also been shown to contribute to improving the expression of key genes in the insulin signaling pathway [59].

In summary, catechins effectively counteract two core pathogenic mechanisms—oxidative stress and chronic inflammation—through synergistic multi-target and multi-pathway actions. The specific research instances in cardiovascular diseases, type 2 diabetes, and neurodegenerative diseases described above establish a solid molecular basis for their chemoprevention against major chronic diseases such as cardiovascular disorders, diabetes, and neurodegenerative conditions.

### 3.5. Synergistic Effects

In the field of nutrition and chronic disease research, the mechanisms of action of single bioactive components have been extensively elucidated. However, the multifactorial and multi-pathway nature of chronic diseases suggests that combined interventions with multiple components may produce synergistic effects through complementary mechanisms and crosstalk. This approach could potentially yield superior preventive and therapeutic outcomes. The four types of components discussed—β-glucan, omega-3 polyunsaturated fatty acids, dietary fiber, and catechins—each possess distinct strengths in anti-inflammation, antioxidant activity, metabolic regulation, and maintenance of gut homeostasis. Their combined application could theoretically establish a multi-level, network-like synergistic defense system.

Chronic low-grade inflammation is a common pathological basis for the vast majority of chronic diseases. These four components can act on different nodes of the inflammatory cascade, producing additive or synergistic inhibitory effects. β-Glucan acts as an immunomodulator. It mildly “trains” the immune system by activating pattern recognition receptors on innate immune cells like macrophages and dendritic cells. This enhances the system’s recognition and response capabilities, creating a more adaptable immune environment. Omega-3 polyunsaturated fatty acids serve as substrates. They give rise to specialized pro-resolving lipid mediators, such as resolvins and protectins. These mediators actively initiate the resolution of inflammation from the upstream, rather than merely suppressing it. Catechins primarily function downstream. They directly block the gene expression of multiple pro-inflammatory cytokines by inhibiting key pro-inflammatory transcription factors like NF-κB and AP-1. SCFAs, fermented from dietary fiber, not only exerts anti-inflammatory effects at the epigenetic level by inhibiting histone deacetylases but also enhances intestinal barrier function. This reduces endotoxin translocation, thereby minimizing the systemic inflammatory triggers at their source. Consequently, the immune “priming” by β-glucan may sensitize immune cells to the anti-inflammatory signals from omega-3 derivatives and the regulatory effects of SCFAs. Omega-3 and catechins cooperatively suppress the inflammatory response from both ends—lipid mediators and intracellular signaling pathways. The SCFAs provided by dietary fiber offer stable gut microenvironmental support for the entire anti-inflammatory network. Studies indicate that combined supplementation may be more effective than single components in reducing systemic inflammatory markers such as C-reactive protein and tumor necrosis factor-α. While the theoretical framework for synergy is well-articulated, the human evidence base remains nascent. Most supporting studies are preclinical. This gap underscores a major research priority: the need for meticulously designed human RCTs that test specific multi-component formulations against their individual constituents to unequivocally demonstrate synergistic efficacy, define optimal ratios, and identify the subpopulations most likely to benefit.

The gut microbiota and its metabolites serve as a central hub linking dietary components to host health, with the synergistic effects of the four components being particularly pronounced at this level. Dietary fiber and β-glucans are classic prebiotics, providing specialized fermentation substrates for beneficial bacteria and promoting the abundant production of SCFAs. This prebiotic effect exhibits high molecular specificity. Research indicates that human gut microbes, particularly members of the Bacteroidetes phylum, have evolved sophisticated genetic systems known as polysaccharide utilization loci (PULs) to recognize and degrade β-glucans of varying chemical structures (e.g., mixed-linkage β-glucans from cereals or β-1,3-glucans from fungi). This means that consuming diverse dietary fibers and β-glucans can selectively stimulate the growth of beneficial microbial taxa harboring the corresponding PULs, thereby directionally optimizing the structure and function of the microbiota. These specifically activated microbial communities efficiently ferment these substrates into SCFAs, function as key synergistic mediators. They not only locally nourish the intestinal epithelium and strengthen the barrier but also enter the systemic circulation to regulate glucose and lipid metabolism as well as immune function in the liver and peripheral organs. Omega-3 fatty acids and catechins can also be metabolized by specific gut microbes into more bioactive forms (e.g., phenolic acid metabolites of catechins), but their efficacy highly depends on a healthy microbial structure.

Insulin resistance and abnormal lipid metabolism are central to metabolic syndrome. Four dietary components exert a multifaceted regulation of glucose and lipid metabolism by targeting multiple physiological stages, including digestion and absorption, hepatic metabolism, and peripheral signaling. In the gastrointestinal tract, β-glucan and dietary fiber form a viscous gel. This physically delays the digestion and absorption of carbohydrates and fats, thereby reducing postprandial peaks in blood glucose and lipids. This physical barrier effect at the “entry point” is supported by molecular-level mechanistic studies. For instance, research on cereal-derived fibers shows that arabinoxylan (AX) and β-glucan (BG), particularly in a 1:1 combination, exhibit significant synergistic effects [61]. They not only entrap starch granules to form a physical barrier, limiting access to water and enzymes, but also bind directly to the active or allosteric sites of α-amylase and α-glucosidase. This binding induces conformational changes in the enzyme proteins via static quenching, directly inhibiting their catalytic activity. This dual mechanism of physical entrapment and biochemical inhibition allows the AX/BG complex to more effectively reduce starch hydrolysis rates and postprandial glycemic responses. It provides concrete molecular evidence for the strategy of “moderating nutrient load at the gastrointestinal front.” The detailed elucidation of the synergistic inhibition of digestive enzymes by arabinoxylan and β-glucan provides a powerful molecular rationale for combining dietary fibers. Nevertheless, the translational success of this specific synergistic combination in human diets remains to be fully quantified. It moves the concept of synergy from a phenomenological observation to a mechanistically explainable interaction, strengthening the scientific foundation for developing multi-fiber functional blends aimed at glycemic control. Omega-3 polyunsaturated fatty acids (PUFAs) primarily act at the transcriptional level. By activating receptors such as peroxisome proliferator-activated receptors (PPARs), they promote fatty acid β-oxidation, inhibit hepatic de novo lipogenesis, and improve adipocyte secretory function. Catechins target the cellular energy sensor. They activate the AMP-activated protein kinase (AMPK) pathway, which promotes glucose uptake and fatty acid oxidation in skeletal muscle while inhibiting enzymes involved in fat synthesis. This cooperation represents comprehensive coverage from the gut to the cell.

In summary, β-glucans, omega-3 polyunsaturated fatty acids, dietary fiber, and catechins demonstrate clear synergistic potential in the prevention and management of chronic diseases. This synergy arises from the deep intersection and reciprocal interactions among four core mechanisms: “anti-inflammatory and immunomodulatory”, “antioxidant defense”, “gut microbiota–metabolism axis”, and “multi-target metabolic regulation”. Their combined effect transcends mere additive outcomes, forming instead a dynamic and multidimensional network of biological effects.

It should be noted that most current evidence of synergy comes from preclinical studies. While this mechanistic and preclinical evidence is compelling, its ultimate public health value and translation into effective strategies depend on a rigorous appraisal of human efficacy, safety, and real-world integration. The following section therefore expands the perspective from molecular and clinical evidence to critically examine the broader health impacts, societal perceptions, regulatory challenges, and future scientific directions for functional foods, thereby connecting mechanistic insights to their practical application and societal relevance [62].

## 4. Functional Foods: Health Impacts, Societal Perspectives and Scientific Advancement

### 4.1. Health Benefits of Functional Foods

Functional foods are conventional foods or modified ingredients with scientifically validated specific physiological modulating functions. Their health value primarily manifests through multidimensional physiological interventions including nutritional optimization, metabolic regulation, gut microbiota modulation, and oxidative stress inhibition. Such foods can effectively supplement essential nutrients (e.g., vitamin D-fortified dairy products), modulate cholesterol metabolism via bioactive compounds like phytosterols, improve gut microbiota ecology using probiotics, and scavenge free radicals through polyphenols. Standardized functional foods demonstrate clear potential in reducing chronic disease risk; however, their application must strictly adhere to scientific principles of safety, efficacy, and dose-response relationships.

β-glucan: Sourced from oats, yeast, and fungi, the health benefits of β-glucan have been robustly validated by science. This component demonstrates significant cardiovascular protection: as a soluble dietary fiber, daily intake of ≥3 g forms a gel-like matrix in the intestine, effectively binding bile acids and promoting their excretion. This thereby drives accelerated hepatic cholesterol metabolism, achieving a 7–24% reduction in LDL-C. Simultaneously, β-(1,3)/(1,6)-glucan enhances innate immune defense by activating the Dectin-1 receptor on macrophages, increasing natural killer (NK) cell activity, and boosting secretion of cytokines such as interleukin-2 (IL-2) and interferon-gamma (IFN-γ). Furthermore, its property of delaying gastric emptying lowers postprandial blood glucose peaks by 20–40%, positively regulating glucose homeostasis.

Omega-3 polyunsaturated fatty acids: Omega-3 fatty acids, represented by EPA and DHA, confer multisystem health benefits. In the cardiovascular domain, EPA reduces triglyceride levels by 10–30% through inhibition of thromboxane A2 (TXA2) synthesis, while DHA decreases ventricular arrhythmia risk by maintaining cardiomyocyte membrane stability. Regarding neurodevelopment, DHA constitutes 15–20% of phospholipids in the cerebral cortex and is essential for neuronal synaptic plasticity; supplementation during pregnancy reduces preterm birth risk by 11% (Cochrane systematic review). When daily intake exceeds 3 g, its metabolite resolvin E1 inhibits the NF-κB inflammatory pathway, significantly improving clinical symptoms of rheumatoid arthritis.

Dietary fiber: Classified by solubility into soluble and insoluble classes, dietary fiber confers systemic protective health effects. In metabolic disease prevention, daily intake of 25–38 g reduces type 2 diabetes incidence risk by 18% (Lancet meta-analysis), a mechanism involving delayed glucose absorption and improved insulin sensitivity. For intestinal health, insoluble fiber (e.g., cellulose) promotes bowel motility by increasing stool volume (elevating defecation frequency by 30%), while soluble fiber (e.g., pectin) is fermented by microbiota to generate short-chain fatty acids such as butyrate, providing energy support for colonic epithelium. Epidemiological studies confirm that for every 7 g increase in daily dietary fiber intake, coronary heart disease risk decreases by 9%.

Catechins: As the core component of green tea polyphenols, epigallocatechin gallate (EGCG) constitutes 60% of total catechins. This compound exerts potent antioxidant effects by scavenging reactive oxygen species and enhancing superoxide dismutase activity through activation of the Nrf2/ARE pathway. At the metabolic regulation level, catechins inhibit intestinal α-glucosidase activity to reduce carbohydrate absorption while promoting fatty acid β-oxidation via the AMP-activated protein kinase (AMPK) pathway (clinical studies show daily supplementation of 400 mg EGCG reduces waist circumference by 3–5 cm). Population-based cohort research (Ohsaki Study) further confirms that individuals consuming >200 mg catechins daily exhibit a 25% significant reduction in cardiovascular disease mortality.

Evidence confirms that functional foods and their core bioactive components promote human health through multi-target mechanisms: β-glucan (≥3 g/day) reduces LDL cholesterol by 7–24% while regulating glucose metabolism and immunity; omega-3 fatty acids (EPA/DHA) lower triglycerides by 10–30% and support neurodevelopment; dietary fiber (25–38 g/day) decreases diabetes risk by 18% and improves intestinal function; catechins (EGCG) reduce cardiovascular mortality by 25% via antioxidant effects and AMPK activation. These benefits are established on specific dosages and standardized application, with their scientific value having gained consensus recognition from international authoritative bodies. However, the pursuit of health benefits through functional foods must be balanced against a prudent consideration of potential risks and an acknowledgment of the current limitations in the evidence base.

### 4.2. Potential Health Concerns of Functional Foods

In contrast to their potential benefits, functional foods are not without risks. Health risks of functional foods primarily stem from ingredient interactions, uncontrolled dosage, and regulatory deficiencies. Excessive intake of fat-soluble vitamins in fortified foods (>3000 μg RE/day) can induce hypervitaminosis A, tripling the risk of hepatic fibrosis. High-dose antioxidant supplementation (>20 mg/day) elevates lung cancer incidence by 16% in smokers. Drug-functional ingredient interactions warrant serious concern: vitamin K-containing functional foods antagonize warfarin’s anticoagulant effect, causing International Normalized Ratio (INR) fluctuations up to 40%. Nutritional imbalances during processing are prominent; for example, oxalates in calcium-fortified juices reduce calcium bioavailability by 35%, while excessive fructose (>50 g/day) in functional candies increases uric acid levels by 19%. Divergent regulatory systems exacerbate risks—for instance, reflecting its stringent evidence requirements, the European Food Safety Authority (EFSA) has been reported to reject approximately 82% of submitted health claim applications [6], whereas some countries lack mandatory safety assessments, enabling a 12.8% market detection rate for weight-loss products illegally adulterated with drug components (e.g., sibutramine).

### 4.3. Critical Analysis of Evidence Limitations and Controversies for Key Bioactive Compounds

An overarching limitation across the functional food components discussed is the disparity between compelling preclinical mechanistic data and the sometimes inconsistent or insufficient evidence from human trials. While the health benefits of functional foods and their bioactive constituents are supported by a growing body of evidence, a critical appraisal reveals significant limitations, inconsistencies, and controversies within the literature for each compound. Moving beyond the general challenges outlined earlier, this section provides a dedicated critical analysis of the evidence gaps and ongoing debates surrounding the key bioactive compounds discussed in this review.

Although numerous meta-analyses confirm the cholesterol-lowering efficacy of β-glucan, the effect size is often modest and exhibits considerable heterogeneity across studies. This variability is influenced by factors such as the food matrix, molecular weight, and viscosity of the β-glucan source. Furthermore, the translation of its immunomodulatory effects, well-documented in in vitro and animal models, into consistent clinical outcomes in humans remains uncertain. Many human trials report high inter-individual variability in immune response, potentially due to differences in baseline immune status, gut microbiota composition, and genetic factors. The highly promising prebiotic and gut-brain axis effects, particularly in neurodegenerative models, are predominantly derived from preclinical studies. Their relevance to human pathophysiology and the requisite effective doses for neuroprotection demand validation through large-scale, long-term human intervention trials.

The most prominent controversy surrounds the cardiovascular and cognitive benefits of ω-3 PUFAs, with large-scale randomized controlled trials (RCTs) and meta-analyses yielding conflicting results. While some studies demonstrate clear benefits for triglyceride reduction and potential neuroprotection, others show null effects on primary endpoints like major adverse cardiovascular events or cognitive decline. This inconsistency is attributed to several factors: the background diet (e.g., ω-6:ω-3 ratio), the dose and chemical form (ethyl ester vs. triglyceride) of the supplement, the baseline ω-3 status of participants, and the stage of disease at which intervention is initiated. The significant effect modification by genetic background, particularly the APOE genotype in Alzheimer’s disease interventions, further complicates the establishment of universal recommendations and highlights the need for stratified approaches in future research.

The relationship between dietary fiber intake and reduced risk of type 2 diabetes is robust in epidemiology; however, intervention studies sometimes show attenuated effects. The metabolic benefits, particularly for insoluble cereal fibers, may not uniformly rely on profound gut microbiota remodeling, suggesting other mechanisms like modulation of protein digestion and amino acid metabolism are at play [47]. This indicates that the prebiotic paradigm does not fully explain the efficacy of all fiber types. Moreover, the efficacy of fiber interventions can be influenced by an individual’s initial gut microbiota composition and genetic makeup. For instance, carriers of the APOE4 allele may derive limited cognitive benefit from fiber-induced microbiota changes, pointing to host genetics as a key modifier of response [49]. High-fiber diets can also cause gastrointestinal discomfort, such as bloating and flatulence, potentially affecting long-term adherence in sensitive individuals.

A major limitation for catechins, particularly epigallocatechin gallate (EGCG), is their inherently low and variable oral bioavailability, which is influenced by factors such as intestinal metabolism, efflux transporters, and food matrix interactions. This raises questions about whether the concentrations required to elicit the potent effects observed in vitro are achievable in vivo through dietary intake [58]. Furthermore, the results from human RCTs are mixed. While some studies report improvements in metabolic parameters and cognitive function, others fail to demonstrate significant benefits. This inconsistency can be attributed to the use of varying catechin sources, doses, formulations, and study durations. High-dose catechin supplements have also been associated with potential hepatotoxicity in rare cases, underscoring the importance of establishing a safe upper intake level. Although innovative approaches like “tea rice” aim to enhance catechin delivery, their long-term safety and efficacy in human populations are yet to be established.

The critical limitations and heterogeneous evidence underscore the necessity of moving beyond generic recommendations toward precision nutrition. The efficacy of functional food components is significantly modulated by inter-individual variability in genetics (e.g., APOE, FADS polymorphisms), baseline health and microbiota status, and disease stage. Future research and product development must embrace a stratified approach, identifying responder subpopulations through integrated multi-omics profiling and well-phenotyped clinical trials. This shift is essential to transform functional foods from broadly promoted commodities into targeted, evidence-based tools for personalized chronic disease prevention.

In conclusion, while the potential of these bioactive compounds is immense, the evidence is not without its caveats. Acknowledging these limitations—including heterogeneous clinical outcomes, inter-individual variability in response, bioavailability constraints, and the gap between preclinical and human evidence—is crucial for the scientific community to design more robust studies and for developing realistic expectations among consumers and policymakers. The future trajectory of the field depends on embracing this complexity, moving beyond universal claims, and focusing on stratified efficacy, personalized delivery, and the translation of elegant mechanisms into consistent human benefits.

### 4.4. Limitations of Animal and in Vitro Data for Human Outcomes

While in vitro and animal studies are indispensable for elucidating molecular mechanisms and providing proof-of-concept, their predictive value for human health outcomes is inherently limited. Several key findings highlighted in this review—particularly the neuroprotective effects of β-glucan and the epigenetic modulatory potential of catechins—rely heavily on such preclinical models, underscoring the need for cautious interpretation and further human validation.

The key limitations in translation include: Cell cultures and animal models cannot fully replicate the complexity of human physiology, chronic disease progression, or the interplay of genetic, environmental, and lifestyle factors that define human responses; Effective doses in animal studies often far exceed achievable human dietary intakes or plasma concentrations, and interspecies differences in absorption, metabolism, and tissue distribution are significant. Animal models of chronic diseases (e.g., Alzheimer’s disease, type 2 diabetes) are typically acute, genetically engineered, or diet-induced approximations that may not capture the slow, multifactorial pathogenesis seen in humans. The gut microbiome, a critical mediator for many functional food components (e.g., β-glucan, fiber), differs substantially between laboratory animals and humans, and its composition is highly individualized. Similarly, human immune responses are more heterogeneous and influenced by prior exposures.

The specific examples in this review illustrate these challenges. The promising neuroprotective synergy between highland barley β-glucan and Lactobacillus, demonstrated in an AD mouse model [21], requires validation in human trials to confirm whether the observed reduction in Aβ deposition and cognitive improvement translates to patients, especially given differences in gut-brain axis wiring and disease chronology. The epigenetic effects of catechins (e.g., EGCG-mediated inhibition of DNMTs and HDACs) are well-documented in cell lines and animal models of cancer and metabolic disease [53,60]. However, whether sustained dietary intake induces similar, functionally significant epigenetic remodeling in human tissues at safe dose levels remains an open question. The molecular-weight-dependent prebiotic effects of β-glucan and the precise anti-inflammatory mechanisms of omega-3 PUFA metabolites, while elegantly shown in in vitro fermentation systems and rodent studies, must be confirmed in human populations with diverse dietary backgrounds and microbiota compositions.

Therefore, while preclinical data provide crucial mechanistic hypotheses and justify clinical investigation, they should not be equated with established human efficacy. The field must prioritize well-designed, long-term human intervention studies—incorporating biomarkers, omics technologies, and stratified designs—to bridge this translational gap and substantiate the preventive potential of functional food bioactives in real-world, human contexts.

### 4.5. Societal Perspectives on Functional Foods

The emergence and development of functional foods are profoundly embedded within the macro-context of contemporary societal transformation and public health needs. Building upon the mechanistic and evidence-based analysis presented in previous sections, this discussion on societal perspectives highlights the critical link between scientific evidence and real-world acceptance. From a societal perspective, their core drivers stem from the convergence of multiple structural factors: intensifying global population aging trends and persistently rising chronic disease burdens impose substantial fiscal and resource pressures on public health systems; simultaneously, pervasive enhancement of public health consciousness has elevated proactive pursuit of disease prevention and health maintenance into a mainstream societal value. Against this backdrop, functional foods have been ascribed a societal role transcending traditional nutritional provision—they are often discussed as a potential component within broader dietary strategies that may contribute to health management and preventive approaches. However, it is important to note that robust economic modeling quantifying their direct impact on alleviating healthcare system burdens at a population level remains an area for further research. Such assessments are necessary to substantiate claims regarding healthcare cost savings and to inform evidence-based public health policy.

Critically, societal acceptance and regulatory paradigms are not monolithic but are directly shaped by the strength, consistency, and translational clarity of the scientific evidence underpinning specific bioactive compounds, as detailed in the preceding sections. This creates a spectrum of credibility that directly impacts consumer trust and regulatory approval. For instance, the cholesterol-lowering efficacy of β-glucan is supported by a robust body of RCTs and meta-analyses [16]. This high-quality, consistent evidence base has directly facilitated authoritative health claims approvals, such as those from the European Food Safety Authority (EFSA). Such clear regulatory endorsement, grounded in strong science, significantly bolsters consumer confidence and market acceptance for β-glucan-enriched products. In stark contrast, the evidence for PUFAs, particularly concerning cognitive benefits and neurodegenerative disease prevention, is more mixed and context-dependent (as outlined in Section 3.2). The efficacy appears modulated by factors such as genetic background (e.g., APOE ε4 carrier status), baseline nutritional status, and intervention stage. This heterogeneity in clinical trial outcomes introduces ambiguity, leading to greater regulatory caution. EFSA, for example, has rejected several cognitive health claims for DHA due to insufficiently conclusive evidence across the general population. This regulatory hesitancy, reflective of the variable evidence, inevitably fuels consumer skepticism and creates a more challenging environment for market acceptance of omega-3 products making cognitive claims.

Similarly, the association between dietary fiber intake, particularly cereal fiber, and a reduced risk of type 2 diabetes is backed by large-scale prospective cohort studies and meta-analyses (e.g., the EPIC-InterAct study, cited in Section 3.3). This strong epidemiological foundation provides a solid basis for public health recommendations and reinforces consumer trust in the value of high-fiber foods. Meanwhile, for compounds like catechins, while the preclinical data on antioxidant and anti-inflammatory mechanisms are compelling, the translation into consistent, long-term human health benefits is still evolving. This evolving evidence base necessitates more nuanced communication to consumers, who may be confronted with exciting mechanistic news but less definitive clinical proof.

Therefore, the variable quality of evidence for different bioactives creates a direct “evidence-trust-regulatory” gradient that shapes the entire functional foods landscape. Consumers with higher health literacy tend to gravitate towards products with robust, human-trial-validated evidence (e.g., β-glucan for cholesterol), while ambiguity (e.g., omega-3 for cognitive health in general populations) fosters caution and scrutiny. Regulatory frameworks, in turn, mirror this gradient, granting claims where evidence is strong and withholding them where it is uncertain.

The food industry, research institutions, and government departments invest substantially in this field to develop scientifically substantiated health-promoting products. However, this development is accompanied by significant societal controversies and challenges: lagging regulatory frameworks, frequent questioning of the sufficiency and generalizability of scientific evidence, potential health inequalities, and excessive marketing that may foster unrealistic consumer expectations of “miracle foods”, thereby overshadowing the fundamental importance of balanced diets and healthy lifestyles. Consequently, societal acceptance of functional foods presents a complex spectrum—they are viewed with interest for their potential role in supporting public health, yet simultaneously provoke extensive debate and cautious scrutiny due to scientific, ethical, and regulatory uncertainties.

Consumer acceptance of functional foods is not homogeneous but exhibits marked heterogeneity and dynamic variability, with its scope profoundly shaped by multiple individual and contextual factors. At the cognitive level, significant disparities exist in consumers’ comprehension and trust regarding functional foods. Consumers with higher health literacy who actively follow nutritional science advancements typically assess product claims and scientific evidence more rationally; their acceptance scope tends to focus on products with robust clinical research support, relatively clear functional mechanisms, and natural or familiar ingredients. Conversely, consumers experiencing information asymmetry, distrusting food industry motives, or holding traditional dietary beliefs may demonstrate heightened vigilance or even rejection. The core boundary of acceptance lies in the strength and credibility of scientific evidence: consumers universally demand efficacy verification—extending beyond corporate marketing—that is provided by independent research institutions and, crucially, validated through human clinical trials. Products lacking such solid evidence or featuring overly broad/vague claims struggle to gain widespread acceptance. Health needs and risk perception are key drivers: individuals with specific health conditions or high-risk profiles show significantly greater acceptance of targeted functional products than healthy populations; simultaneously, consumers meticulously weigh perceived health benefits against potential risks and costs. Furthermore, information source credibility, product attributes, cultural background, label clarity, and regulatory safeguards collectively form a complex decision-making network. Overall, consumer acceptance constitutes a dynamic “trust-value” evaluation interval: its upper limit is determined by the robustness of scientific evidence and the salience of health benefits, while its lower boundary is defined by concerns regarding safety, transparency, and value-for-money. Currently, consumers generally maintain a “cautiously open” stance toward functional foods—actively seeking health solutions while remaining highly vigilant toward insufficient scientific substantiation and overmarketing—which presents the primary challenge for market expansion.

### 4.6. Scientific Advancement of Functional Foods

The development of functional foods presents a landscape interwoven with significant advantages and complex challenges. Its core driver lies in delivering specific health benefits beyond basic nutrition through scientifically validated bioactive substances, meeting the growing health management demands of modern populations while creating high-value-added growth pathways for the food industry. Nevertheless, the sector faces multifaceted challenges: significant divergence in global regulatory frameworks creates complex market access rules and inconsistent claim standards; technical bottlenecks persist in ensuring the stability and bioavailability of functional ingredients during processing and storage; establishing solid evidence-based medical support for health claims requires long-term and costly scientific investment; consumer knowledge gaps and information overload further impede market education.

Current development trends focus on personalized nutrition solutions integrated with advanced technologies—such as utilizing genomics, microbiomics, and artificial intelligence for precision product design; in-depth exploration of novel functional components like ingredients with dual food-medicine properties, microbial metabolites, and phytochemicals; deepening application of digital tools in consumer engagement and personalized recommendations; while sustainability and clean label demands profoundly influence raw material selection and production processes. However, these approaches, while promising, often remain in the proof-of-concept or early-stage clinical validation phase.

Looking forward, the functional food market demonstrates substantial potential amid population aging, chronic disease prevention, and health-conscious consumption upgrading. Its sustainable development critically depends on deep interdisciplinary collaboration, international regulatory harmonization to facilitate equitable trade, continuous innovation ensuring product efficacy and safety, and transparent consumer communication to build trust. Long-term success will be determined by a dynamic equilibrium among scientific rigor, regulatory adaptability, and genuine market needs. A summary comparing the strength of evidence for the four key bioactive compounds is provided in Table 4.

## 5. Conclusions

Chronic diseases, characterized by high prevalence, prolonged duration, and cumulative health damage, constitute a core burden on global public health systems. This review, through its unique “mechanism-evidence-regulation” framework, has systematically synthesized the scientific and translational landscape of functional foods. Underpinned by the food-medicine homology theoretical framework, this review systematically analyzes the multidimensional mechanisms and evidence-based support for functional foods in targeted chronic disease prevention. Research demonstrates that specific functional food components modulate core pathological processes—including oxidative stress, chronic inflammation, metabolic dysregulation, and gut microbiota imbalance—forming a synergistic intervention network at molecular, cellular, and systemic levels. This provides scientifically viable nutritional strategies for preventing cardiovascular diseases, type 2 diabetes, and neurodegenerative disorders.

The proposed efficacy mechanisms of key bioactive components, as supported by evidence ranging from molecular to clinical levels, are summarized as follows:

1. β-Glucan (daily intake ≥ 3 g) significantly reduces LDL-C by 0.25–0.30 mmol/L through viscous gel formation and bile acid binding (meta-analysis evidence). Its prebiotic properties drive colonic microbiota to produce short-chain fatty acids (SCFAs), mediating systemic anti-inflammatory and endothelial protection via GPR41/43 receptors; high-molecular-weight variants (e.g., HMW barley β-glucan) further optimize microbial structure, directly correlating with improved cardiovascular risk markers.
2. Omega-3 PUFAs (EPA/DHA) structurally integrate into neuronal membranes (DHA constitutes 15–20%) and regulate neuroprotective pathways through metabolites like neuroprotectin D1 (NPD1), inhibiting Aβ aggregation and Tau phosphorylation (39% reduction in animal models). Their anti-inflammatory mechanism involves blocking NLRP3 inflammasome activation and pro-inflammatory cytokine release, exhibiting genetic background dependency in early Alzheimer’s intervention (significant benefits in APOEε4 non-carriers).
3. Dietary Fiber (daily intake 25–38 g) delays glucose absorption via physical barrier effects and activates AMPK/SIRT1 longevity pathways through SCFAs (propionate/butyrate), improving insulin sensitivity (HbA1c reduction 0.5–1.0%). The unique value of insoluble cereal fiber is supported by large-scale cohort studies.
4. Catechins (EGCG) enhance endogenous antioxidant defense via the Keap1-Nrf2/ARE pathway and suppress NF-κB nuclear translocation to reduce inflammatory mediator expression. Their epigenetic regulation (DNMTs/HDACs inhibition) reverses metabolic gene silencing; clinically, >500 mg daily EGCG intervention significantly improves insulin resistance and vascular endothelial function (Ohsaki Study: 25% cardiovascular mortality reduction).

Current functional food development demonstrates precision and scientific refinement: multi-omics technologies drive personalized nutrition design; innovations in delivery systems enhance bioactive bioavailability; regulatory frameworks strengthen requirements for high-level evidence. Nevertheless, persistent challenges include ingredient stability control, standardization of dose-response relationships, validation of efficacy generalizability across populations, and international regulatory harmonization. As critically evaluated in Section 4.4, while preclinical studies provide indispensable mechanistic insights, a key translational gap remains for several promising effects (e.g., neuroprotection via the gut-brain axis, epigenetic modulation). Future research must prioritize human trials to validate these mechanisms and establish efficacy.

## 6. Perspectives and Outlook

Building upon the synthesis presented in this review, the field must now prioritize closing critical knowledge gaps through hypothesis-driven research. We propose the following specific and testable hypotheses as clear directions for future investigation:

The neuroprotective efficacy of combined highland barley β-glucan and *Lactobacillus* is contingent upon the specific induction of gut bacteria capable of producing the SCFA butyrate (e.g., *Faecalibacterium prausnitzii*), and the subsequent upregulation of brain-derived neurotrophic factor (BDNF) in the hippocampus, with this effect being significantly attenuated in animal models upon pharmacological inhibition of GPR41/43 receptors. A mechanistic understanding of how specific probiotic-prebiotic synergies translate gut microbial metabolic output into precise neuroprotective signals.

The lipid-lowering response to oat β-glucan and the cognitive benefit from DHA supplementation exhibit significant interaction, such that individuals with a “high-bile-acid-loss” enterotype and the APOE ε4 allele derive superior combined benefit, attributable to the normalization of a shared disrupted gut-liver-brain axis. The development of integrated host-genetic and microbiome-based models to predict individual responsiveness to multi-component functional food regimens, moving beyond one-size-fits-all recommendations.

Acid-hydrolyzed, low-molecular-weight β-glucans from Pleurotus tuber-regium (PTR-HBG) will demonstrate superior prebiotic selectivity and SCFA production in vitro compared to native polymers, and this in vitro advantage will directly translate to a greater reduction in atherosclerosis progression in ApoE^−/−^ mouse models, specifically by enriching for *Bifidobacterium* spp. that enhance gut barrier integrity. Establishing a direct causal chain from controlled structural modification of bioactives, to targeted shifts in microbial ecology, and finally to definitive health outcome measures in relevant disease models.

Long-term supplementation with EGCG (>500 mg/day for 6 months) in prediabetic subjects will lead to measurable demethylation of the promoter region of the *NRF2* gene in peripheral blood mononuclear cells, and the degree of demethylation will be positively correlated with improvements in HOMA-IR and serum 8-OHdG levels, providing a direct molecular link between catechin intake, epigenetic remodeling, and metabolic health. A critical lack of human evidence demonstrating that the promising epigenetic effects of food bioactives observed in vitro and in animal models are operational and clinically relevant in human populations.

To bridge mechanistic insights and clinical application, a focused research framework is proposed below (Table 5).

This concise framework prioritizes testable hypotheses across core mechanisms, aim-ing to develop stratified interventions and validate biomarkers for personalized nutrition.

Addressing these priorities will require a concerted effort integrating controlled laboratory studies, well-designed human trials with deep phenotyping, and the application of systems biology approaches. The ultimate goal is to transition from generic health claims to the development of functionally validated, personalized food solutions with clearly elucidated and measurable mechanisms of action.

Future advancement necessitates large-scale cohort studies and real-world evidence to elucidate “food-microbiota-host” interaction mechanisms, thereby promoting high-value transformation of food-medicine homology resources. Future research should also prioritize elucidating the specific mechanisms underlying synergistic interactions among different functional food components. Translating these insights into effective, personalized multi-component nutritional regimens will be crucial for advancing the field.

In summary, functional foods provide safe and efficacious dietary strategies for chronic disease primary prevention through multi-target, network-based intervention paradigms. Their successful translation relies on interdisciplinary collaboration, stringent regulatory oversight, and enhanced public scientific literacy—ultimately enabling a paradigm shift from broad-spectrum health maintenance to precision chronic disease prevention.

## Figures and Tables

**Table 1:** Comparative profile of key bioactive compounds: mechanisms, disease targets, and personalization factors.

Bioactive	Main Mechanisms	Primary Disease Target	Key Personalization Factors
β-Glucan	LDL-R, SCFA, prebiotic	Cardiovascular disease	MW, gut microbiota
Omega-3 PUFAs	Neuroprotection, anti-inflammation	CVD, neurodegeneration	APOE genotype, baseline status
Dietary Fiber	SCFA, AMPK, glycemic control	Type 2 diabetes	Fiber type, microbiota
Catechins	Nrf2/ARE, NF-κB inhibition	CVD, metabolic disease	Bioavailability, matrix

**Table 2:** Key factors contributing to inconsistent outcomes in clinical trials of Omega-3 PUFAs.

Factor Category	Key Implication
Baseline Nutritional & Health Status (e.g., low ω-3 levels) [32]	Greater benefit observed in deficient populations; “ceiling effect” in replete individuals.
Genetic Factors (APOE ε4, FADS polymorphisms) [36]	APOE ε4 carriers show diminished benefit; FADS variants affect endogenous synthesis efficiency.
Trial Design (dose, form, duration)	Contributes to inconsistent findings and effect heterogeneity.
Disease Stage (prevention vs. treatment)	Intervention in early/preclinical stages yields greater efficacy than in advanced disease.

**Table 4:** Summary of evidence strength for key functional food bioactives in chronic disease prevention.

Bioactive Compound	Preclinical Evidence	Small Clinical Trials	Large RCTs/Meta-Analyses	Overall Evidence Strength
β-Glucan	Strong (mechanistic)	Consistent (lipid/glycemia)	Strong (confirmed LDL-C reduction)	High for CVD risk reduction
Omega-3 PUFAs	Strong (neuro/anti-inflammatory)	Inconsistent (cognitive/CVD)	Mixed (context-dependent)	Moderate, context-dependent
Dietary Fiber	Strong (SCFA, metabolism)	Supportive (glycemia/satiety)	Strong (consistent T2DM risk)	High for T2DM prevention
Catechins	Strong (antioxidant, epigenetic)	Supportive but variable	Limited (few large-scale RCTs)	Moderate, promising

**Table 5:** Proposed hypotheses and research framework for advancing functional food bioactives in personalized nutrition.

Hypothesis	Mechanistic Focus	Model	Translation
Highland barley β-glucan + *Lactobacillus* synergy requires butyrate & GPR41/43. [20]	Gut-Brain Axis	AD mice; FMT.	Synbiotic for cognitive health.
Oat β-glucan + DHA most effective in “high-bile-acid-loss” APOE ε4 carriers.	Host-Gut Interaction	Human RCT; APOE ε4 mice.	Predictive model for dementia prevention.
Engineered low-MW β-glucan (PTR-HBG) superior to native polymer [13].	Structure-Function	In vitro fermentation; *ApoE*^−/−^ mice.	Optimized prebiotic ingredient.
EGCG induces *NRF2* demethylation, correlating with metabolic improvement [51].	Epigenetics	Human trial (prediabetes).	Biomarker for efficacy.
AX/BG (1:1) synergy inhibits digestive enzymes, reducing glycemia.	Nutrient Absorption	In vitro assay; human meal study.	Multi-fiber blend for glycemic control.

## Data Availability

No new data were created or analyzed in this study.
